# Change‐of‐function fibroblast growth factor receptor 3 mutations causing skeletal dysplasias

**DOI:** 10.1002/ccs3.70119

**Published:** 2026-10-30

**Authors:** Tanaya Roy, Zuzana Feketova, Jiawei Liu, Pavel Krejci, Kalina Hristova

**Affiliations:** ^1^ Department of Materials Science and Engineering and Institute for NanoBioTechnology Johns Hopkins University Baltimore Maryland USA; ^2^ Department of Biology Faculty of Medicine Masaryk University Brno Czechia; ^3^ International Clinical Research Center St. Anne's University Hospital Brno Czechia; ^4^ Institute of Animal Physiology and Genetics of the Czech Academy of Sciences Brno Czechia

**Keywords:** achondroplasia, cell signaling, Crouzon syndrome, FGF, fibroblast growth factor receptor 3, ligand bias, membrane protein, mutation, phenotype

## Abstract

Fibroblast growth factor receptor 3 (FGFR3) is a membrane protein that plays a critical role as a negative regulator in the growth and development of the skeletal system. Mutations in FGFR3 cause a range of skeletal dysplasias. The G380R mutation in FGFR3 causes achondroplasia (ACH), the most common form of human short‐limbed dwarfism, whereas the A391E mutation in FGFR3 causes Crouzon syndrome, a craniosynostosis characterized by premature fusion of sutures between the cranial bones, with acanthosis nigricans. Both mutations are considered “gain‐of‐function” and occur close to each other within the simple FGFR3 helix that spans the membrane. Yet, the two pathological phenotypes of ACH and Crouzon syndrome are distinctly different. Here, we show that FGFR3 engages in biased signaling. We further show that the G380R and A391E mutations do not simply amplify signaling but differentially reshape the FGFR3 signaling patterns. The data provide an explanation of the distinctly different phenotypes due to the two mutations and lead us to propose that the mutations are “change‐of‐function.”

## Graphical abstract

We show that the ligands FGF1 and FGF2 elicit differential fibroblast growth factor receptor 3 (FGFR3) signaling responses and that the FGFR3 G380R and A391E mutations reshape these signaling outputs. Thus, pathogenic FGFR3 mutations found in skeletal dysplasia do not simply enhance signaling strength and should be reclassified from “gain‐of‐function” to “change‐of‐function” mutations.

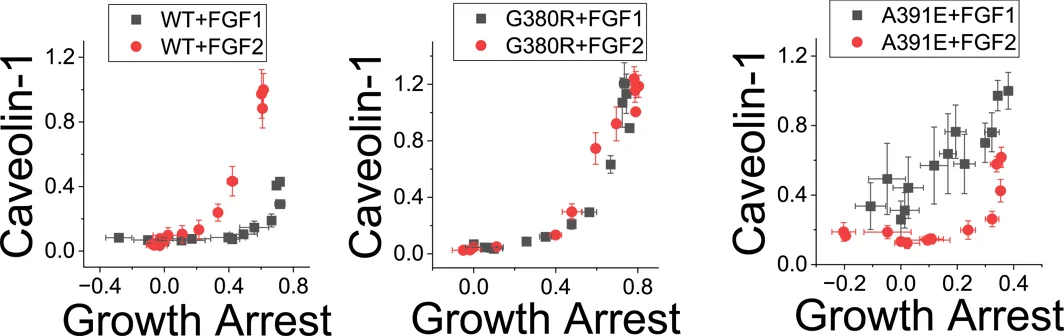

## 1 INTRODUCTION

The fibroblast growth factor (FGF) family consists of 22 members with mitogenic or metabolic activities.^1^, ^2^, ^3^ FGFs signal through four FGFR receptors, FGFR1 to FGFR4,^4^, ^5^, ^6^ which are essential for virtually all tissue and organ development and for maintenance of tissue homeostasis.^1^, ^7^ FGFRs play a particularly important role in the development of the skeletal system, which encompasses more than 200 bones in the adult body, including the long bones and the skull.^8^, ^9^

In the 1990s, the importance of FGFR signaling in skeletal system development became clear after researchers identified more than 100 mutations in FGFR1, FGFR2, and fibroblast growth factor receptor 3 (FGFR3) that cause skeletal dysplasias affecting the long bones and skull.^7^, ^10^, ^11^, ^12^, ^13^ These genetic discoveries inspired basic research into FGFR signaling in vitro and led to identification of key components of FGFR signaling pathways.^14^, ^15^, ^16^, ^17^, ^18^, ^19^, ^20^ Numerous FGFR mutations were studied and found to cause either over‐activation or inhibition of key signaling pathways.^10^, ^11^, ^21^, ^22^, ^23^, ^24^, ^25^, ^26^

One of the receptors, FGFR3, typically inhibits proliferation and serves as a negative regulator of bone growth.^27^ Mutations in FGFR3 cause well‐known skeletal dysplasias.^12^, ^28^, ^29^, ^30^, ^31^ The most extensively studied FGFR3 mutation is G380R, which causes achondroplasia (ACH),^11^, ^32^ characterized by disproportionate short stature with particularly short arms and legs, and a large head with a prominent forehead.^33^, ^34^ In contrast, the A391E substitution in FGFR3 causes Crouzon syndrome, a craniosynostosis characterized by poor growth of sutures between the cranial bones, resulting in premature suture fusion, defective shape of the cranium and face, bulging eyes, small nose, and underdeveloped upper jaw.^14^, ^24^, ^35^ Acanthosis nigricans occurs with Crouzon syndrome and is manifested in rough dark skin patches. Importantly, ACH is a dwarfing condition, in contrast to Crouzon syndrome. Conversely, the craniosynostosis and acanthosis nigricans typical of Crouzon syndrome do not occur in ACH.

Both the ACH (G380R) and the Crouzon (A391E) mutations are in the transmembrane domain of FGFR3, and they are positioned only 10 amino acids from each other within the simple helix that spans the membrane. Yet, these two mutations cause two vastly different pathophysiologic phenotypes with minimal overlap, a paradox that has remained unresolved thus far.

Here, we sought to characterize FGFR3 signaling in rat chondrosarcoma (RCS) chondrocytes, an immortal chondrocyte cell line, which is a well‐characterized cellular model for FGFR3‐related skeletal dysplasias. FGFR3 is expressed in chondrocytes in the growth plate of long bones and controls their proliferation and differentiation,^36^ which are disturbed in ACH.^29^, ^30^, ^33^, ^34^ FGFR3 has been reported to be expressed in cranial cartilage, including the cartilage plate that underlies the coronal sutures.^37^ Recent work in zebrafish has demonstrated that FGFR3 is crucial for regulating osteogenesis within these sutures.^38^ Importantly, FGFR3 signaling in cartilage can be reproduced in vitro in RCS cells.^39^, ^40^ RCS chondrocytes respond to FGF‐mediated activation of endogenous FGFR3 via growth arrest that depends on extracellular signal‐regulated kinase signaling and produce lamin A/C and caveolin‐1 as senescence markers.^39^ Recently, we generated the RCS_Fgfr1‐4 *null* cells, where all four endogenous *FGFR* genes were disrupted using clustered regularly interspaced short palindromic repeats/Cas9.^41^ For this work, RCS_Fgfr1‐4 *null* cells were stably transfected with plasmids encoding the human wild‐type (WT) FGFR3, the G380R‐FGFR3, and the A391E‐FGFR3.

Here, we compared the action of two ligands, FGF1 and FGF2, because they are important for chondrocyte signaling and for development of the long bones and the skull.^3^, ^7^, ^42^, ^43^ Both ligands are expressed in growth plates of long bones (affected in ACH), but their expression is distinctly different.^36^ FGF2 is expressed in the proliferative chondrocyte zone, whereas FGF1 is expressed in the hypertrophic chondrocyte zone.^36^ In the developing fetal skull, FGF1 is expressed in the dura mater (the connective tissue just under the skull) during formation of the sutures.^44^ By the time the fetus is completely developed, however, FGF2 has replaced FGF1 in this location. Previous work has shown that, although FGF1 induces suture obliteration and fusion more effectively than FGF2, FGF2 is the more potent inducer of the gene encoding the transcription factor Msx2.^45^ This prior work suggests that FGF1 and FGF2 exhibit different activities in vivo and instruct cells during different stages of development. We thus explored the possibility that FGF1 and FGF2 are biased ligands when signaling via FGFR3 and that the mutations alter the bias.

## 2 RESULTS

By definition, “ligand bias” means the ability of ligands to differentially activate different signaling pathways downstream of the same receptor.^46^ The workflow to study signaling bias was first developed to study GPCRs^47^ and later shown to be applicable for RTKs.^48^ Following these protocols, we used RCS cells stably expressing human WT FGFR3 to measure dose–response curves, while varying the concentration of ligands FGF1 and FGF2. One important functional response of RCS cells is the growth arrest (an antiproliferation marker) induced by the two ligands, FGF1 and FGF2 (Figure 1A). Cell‐counting experiments after 72 h of ligand treatment demonstrated that the cell count decreased as the concentrations of FGF1 and FGF2 were increased. We plotted the relative decreases as “growth arrest” in Figure 1A. The functional form of the growth‐arrest response is a rectangular hyperbola, as expected, with a plateau level indicating the efficacy of the ligand. The efficacy of FGF1 in inducing growth arrest is higher than the efficacy of FGF2.

**FIGURE 1:**
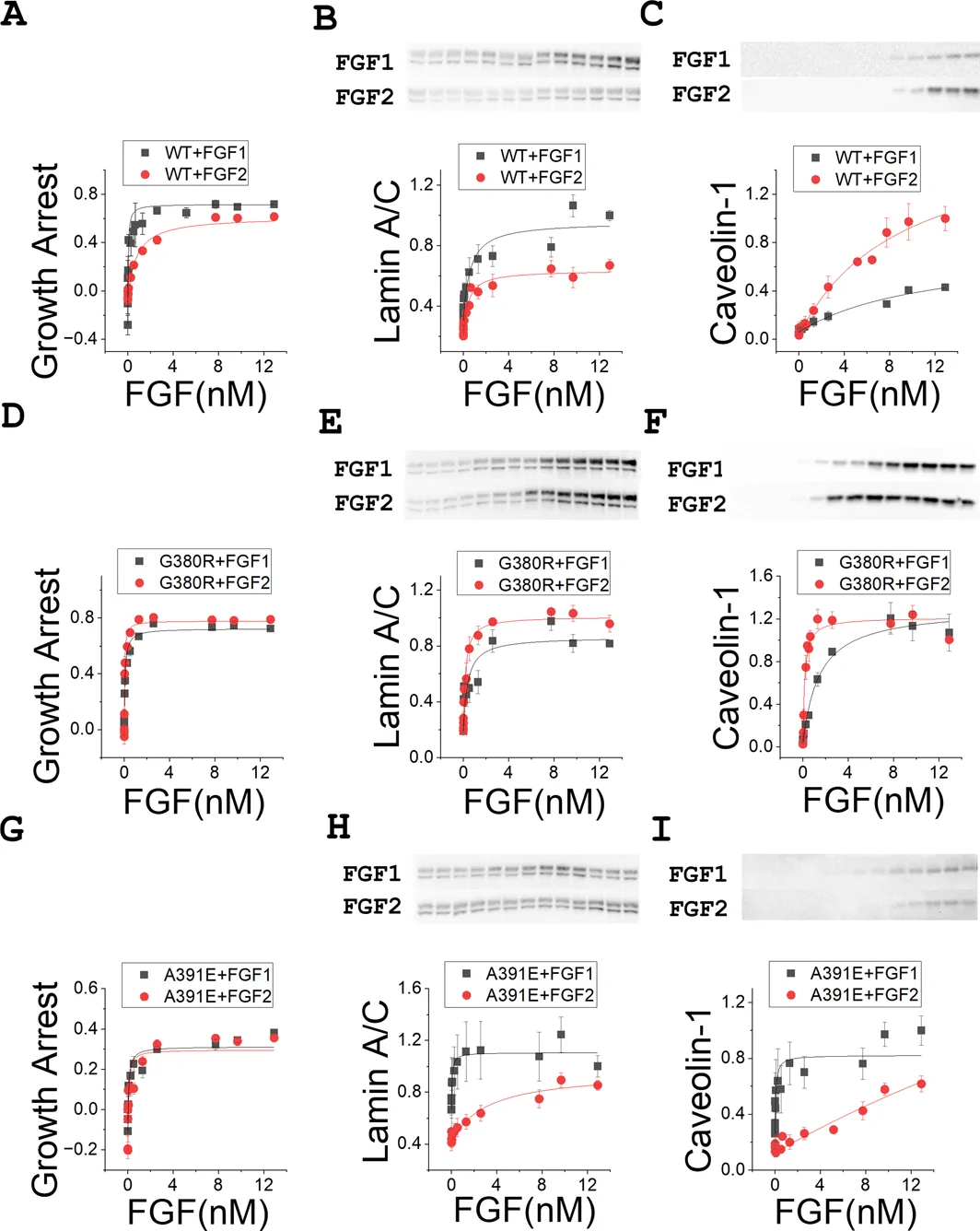
Dose–response curves for WT‐FGFR3 (A–C), G380R‐FGFR3 (D–F), and A391E‐FGFR3 (G–I), collected while varying the concentrations of the ligands FGF1 and FGF2 from 0 to 15 nM. When Western blot was used for quantification, a representative blot is shown above the dose–response curves. Dose–response curves are the averages from three independent biological experiments.

We next used Western blot to measure levels of lamin A/C, a known senescence marker, over the concentration range used in the growth‐arrest measurements, after 24 h of ligand treatment. Again, the efficacy of FGF1 to induce this response is higher than the efficacy of FGF2, but the difference in efficacies of the two ligands is larger than in the growth‐arrest case (Figure 1B). We next used Western blotting to measure levels of caveolin‐1, another senescence marker, over the concentration range used in the growth‐arrest measurements. In this case, FGF2 appears to have higher efficacy than FGF1, so the efficacy rank order is reversed (Figure 1C).

These differences in dose–response curves indeed imply that FGF1 and FGF2 are biased ligands when signaling through FGFR3. To visualize their preferences for inducing one response over another, we used the dose–response curves to construct bias plots as a graphical representation of the magnitude and direction (sign) of the preferences.^49^ A bias plot is created by plotting the magnitude of one response (the *y*‐axis value of the dose–response curve) against the magnitude of the second response (the *y*‐axis value of the second dose–response curve) for each ligand concentration (i.e., for the same *x*‐axis value of the dose–response curves).^50^ The bias trajectories for different ligands can be directly compared in a bias plot.^49^ The bias plots comparing the three responses are shown in Figure 2A–C. There is no bias (no difference in preference for one response over another when comparing the two ligands) in Figure 2A. However, in Figure 2B,C, we see that FGF1 exhibits a preference toward caveolin‐1 induction (on the *y*‐axis) and against growth arrest and lamin A/C induction (on the *x*‐axis). On the other hand, FGF2 is biased toward growth arrest and lamin A/C and against caveolin‐1.

**FIGURE 2:**
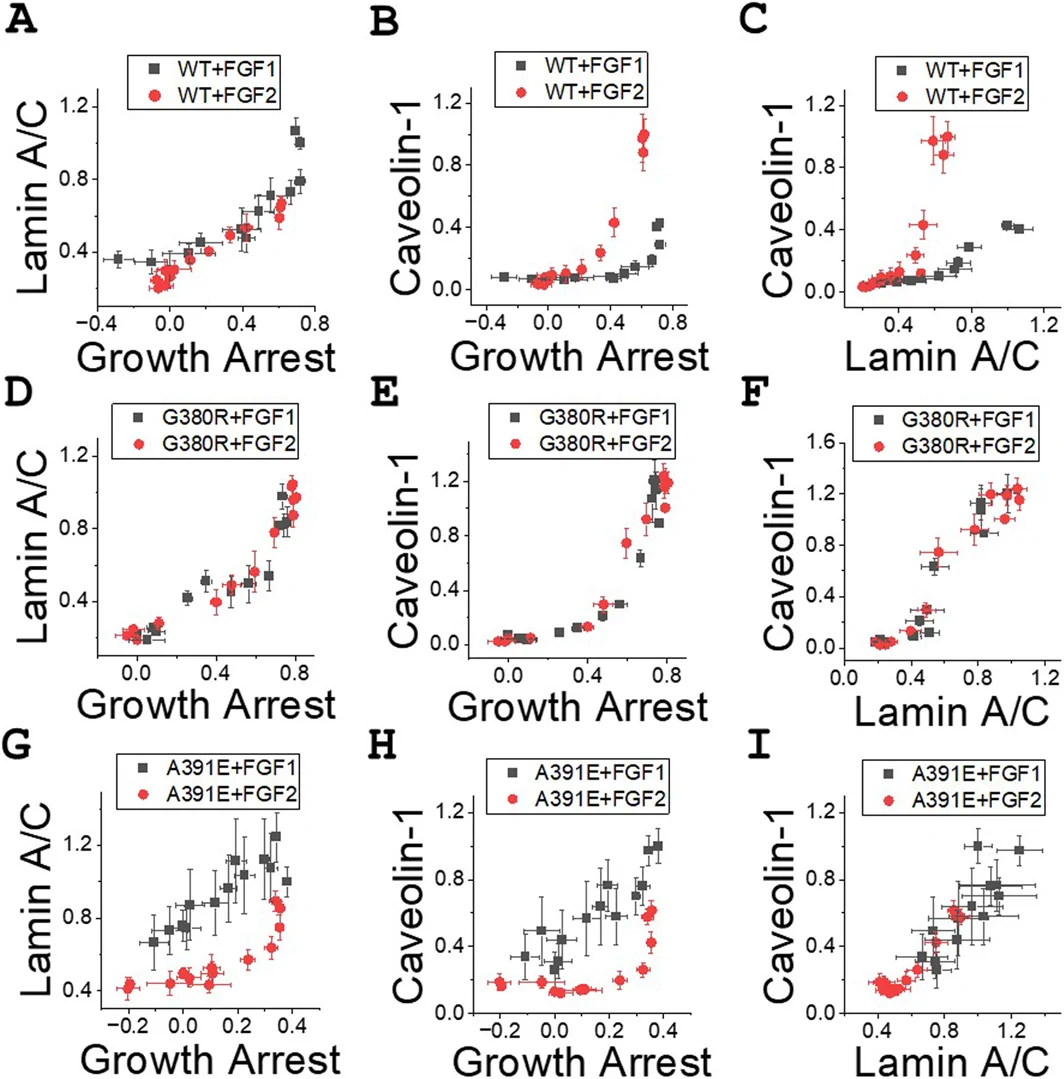
Bias plots reporting on the preferences of the two ligands, FGF1 and FGF2, for downstream responses. Bias plots are shown for wild‐type FGFR3 (A–C), G380R‐FGFR3 (D–F), and A391E‐FGFR3 (G–I), such that the preferences of the two ligands for the downstream responses when signaling through the three FGFR3 variants can be compared within each column of bias plots. For instance, comparisons of panels (B, E, H) (middle column) show that the ligand bias observed in WT FGFR3 is lost in the G380R mutant and is reversed in the case of the A391E mutant.

We also collected dose–response curves with G380R‐FGFR3 (Figure 1D–F) and A391E‐FGFR3 RCS cells (Figure 1G–I). Bias plots constructed from these dose–response curves for G380R‐FGFR3 are shown in Figure 2D–F, and bias plots for A391E‐FGFR3 are shown in Figure 2G–I.

Now we can compare the preferences of the two ligands in inducing a pair of responses when signaling through WT, G380R‐, and A391E‐FGFR3. Comparison of Figure 2A,D,G (the first column of the bias responses in Figure 2, comparing lamin A/C and growth arrest) shows that the two ligands are biased only when signaling through A391E‐FGFR3: FGF1 preferentially induces lamin A/C expression, whereas FGF2 preferentially induces growth arrest. Comparison of Figure 2B,E,H (the second column of the bias responses in Figure 2, comparing caveolin‐1 and growth arrest) shows that the two ligands are biased when signaling through WT FGFR3 and A391E‐FGFR3 but not through G380R‐FGFR3. Remarkably, the preferences of the two ligands are opposite when signaling through WT FGFR3 versus A391E‐FGFR3. Comparison of Figure 2C,F,I (the third column of bias responses in Figure 2 comparing caveolin‐1 and lamin A/C) shows that the two ligands are biased only when signaling through WT FGFR3.

We thus see that the preferences of FGF1 and FGF2 for FGFR3 downstream responses are scrambled when they signal through the mutants. The effects of the two mutations are distinctly different. Thus, the mutations should be categorized as “change‐of‐function” rather than as gain‐ or loss‐of‐function, as they do not simply amplify or inhibit signaling.

## 3 DISCUSSION

In 2021, advances in elucidating the molecular pathology of ACH resulted in a clinical therapy when the C‐natriuretic peptide analog vosoritide (Voxzogo) was approved to treat ACH.^51^, ^52^, ^53^, ^54^ Several other treatments are currently in final stages of clinical evaluation for ACH.^55^ However, there are no treatments in development for Crouzon syndrome associated with FGFR3 mutations, or for similar syndromes caused by mutations in FGFR1 and FGFR2. This is largely because our understanding of the pathological mechanisms underlying FGFR‐driven craniosynostoses has remained poor. We do not know which factors contribute to the array of phenotypes observed in these syndromes, such as craniosynostosis, syndactyly, macrocephaly, and midfacial hypoplasia. Here, we showed that the G380R and A391E mutations are “change‐of‐function” mutations, as they do not simply amplify or inhibit signaling but differentially reshape the outcome of FGFR3 signaling. Our data suggest that FGFR3 response to FGF ligands in Crouzon syndrome is distinctly different from that in ACH and provide a possible explanation for the marked differences in phenotypes caused by these two mutations. Drugs that are effective for ACH will likely not work for Crouzon syndrome, which appears to be caused by different patterns of FGFR3 signaling. The presented work is the first step toward understanding differential FGFR3 signaling in ACH versus Crouzon syndrome. We hope that it will inspire future basic work that could lead to new therapies to treat Crouzon syndrome.

## AUTHOR CONTRIBUTIONS

**Tanaya Roy**: Investigation; data analysis; writing original draft. **Zuzana Feketova**: Investigation; cell model generation. **Jiawei Liu**: Investigation. **Pavel Krejci**: Conceptualization; supervision; funding acquisition; manuscript editing. **Kalina Hristova**: Conceptualization; supervision; funding acquisition; manuscript editing.

## CONFLICT OF INTEREST STATEMENT

The authors declare no conflicts of interest.

## ETHICS STATEMENT

None.

## Supporting information

Supporting Information S1

Supporting Information S2

## Data Availability

The data that support the findings of this study are available from the corresponding author upon reasonable request.
