# Supplementary material for: Change‐of‐function fibroblast growth factor receptor 3 mutations causing skeletal dysplasias
Source: J Cell Commun Signal. 2026 Oct 30;20(4):e70119. doi: 10.1002/ccs3.70119 (PMC13722780; doi:10.1002/ccs3.70119)
Supplement: Supplementary file 1 [file CCS3-20-e70119-s001.pdf]

<u>RCS cells used in the manuscript:</u>
species rat,
sex N/A
tissue of origin chondrosarcoma
official cell line name RCS
Research Resource Identifier (RRID) CVCL_S122

source/supplier of the cell line and when it was obtained have been included
RCS cells were established in 1996 by Krish Mukhopadhyay, Veronique Lefebvre, Guang Zhou, Silvio Garofalo, James H. Kimura, Benoit de Crombrugghe; *Use of a new rat chondrosarcoma cell line to delineate a 119-base pair chondrocyte-specific enhancer element and to define active promoter segments in the mouse pro-alpha 1(II) collagen gene*. J. Biol. Chem. 270:27711-27719(1995)

Krejci lab acquired the cells from Benoit de Crombrugghe in 2002; cell lines used in the article were developed in Krejci lab.

Confirmation that the cell line was authenticated for the described experiments YES
has not been previously reported as misidentified or contaminated Not reported misidentified or contaminated

Please confirm that the cell line was free of mycoplasma contamination for the described experiments. Free of mycoplasma
