# Supplementary material for: Change‐of‐function fibroblast growth factor receptor 3 mutations causing skeletal dysplasias
Source: J Cell Commun Signal. 2026 Oct 30;20(4):e70119. doi: 10.1002/ccs3.70119 (PMC13722780; doi:10.1002/ccs3.70119)
Supplement: Supplementary file 2 [file CCS3-20-e70119-s002.docx]

**SUPPLEMENT**

**Change-of-function FGFR mutations causing skeletal dysplasias**

*Tanaya Roy^a^, Zuzana Feketova^b,c^, Jiawei Liu^a,^ Pavel Krejci^b,c^ , and Kalina Hristova^a^*

^a^Department of Materials Science and Engineering and Institute for NanoBioTechnology, Johns Hopkins University, Baltimore MD, USA

^b^Department of Biology, Faculty of Medicine, Masaryk University, and International Clinical Research Center, St. Anne's University Hospital, Brno, Czechia

^c^Institute of Animal Physiology and Genetics of the Czech Academy of Sciences, Brno, Czechia

**Material and Methods**

Cell culture

RCS cells expressing human FGFR3c variants were generated from wild-type RCS cells in which the endogenous *Fgfr1-4* genes had been inactivated using CRISPR-Cas9 (RCS_Fgfr1-4 *null* cells).^42^ RCS_Fgfr1-4 *null* cells were stably transfected with full-length human wildtype (WT) FGFR3c, G380R-FGFR3c (Addgene #214908) or A391E-FGFR3c (Addgene #255932) and a GFP reporter using PiggyBac transposase. The WT FGFR3c and G380R-FGFR3c cells had been described previously^57^, while the A391E-FGFR3c cells were generated for this study using the same strategy. RCS cells were grown in DMEM media supplemented with 10% FBS (Gibco) and antibiotics (Invitrogen). Cells were seeded into 24-well tissue culture plates (TPP) at a density of 10,000 cells/well, grown for 24 hours, treated with FGF1 (232-FA-025; RnD Systems) or FGF2 (233-FB-025; RnD systems) in the presence of heparin (1 µg/µl; Sigma) for 72 hours, and counted. For each biological replicate, cells were then manually counted two times and the results were averaged. Three biological replicates were analyzed for each condition.

Cell authentication

Cells were authenticated by commercial short tandem repeat fragment analysis (Microsynth AG). Briefly, highly polymorphic short tandem repeat loci analysis was done on the ABI3730xl DNA Analyser (Life Technologies) and the resulting data were analyzed with GeneMarker software (Softgenetics).

Western blot

RCS cells were seeded into 24-well plates at a seeding density of 1x10^5^ cells/well, grown for 24 hours and treated with FGF1 or FGF2 in the presence of heparin for 24 hours. Three biological replicates were analyzed for each condition. Cells were lysed in Laemmli sample buffer (BioRad). Lysates were resolved by SDS-PAGE and transferred to a PVDF membrane. The primary antibodies used were Lamin A/C rabbit polyclonal antibody (2032S; Cell Signaling Technology), and Caveolin-1 rabbit polyclonal antibody (3238S; Cell Signaling Technology). Western blot signals were visualized by chemiluminiscence (34095; Thermofisher). Bio-Rad GelDoc XRS+ was used to image the membrane. Densitometric analysis of the blots were performed using ImageJ.
